# Specificity of the innate immune responses to different classes of non-tuberculous mycobacteria

**DOI:** 10.3389/fimmu.2022.1075473

**Published:** 2023-01-18

**Authors:** Wanbin Hu, Bjørn E. V. Koch, Gerda E. M. Lamers, Gabriel Forn-Cuní, Herman P. Spaink

**Affiliations:** Institute of Biology, Leiden University, Leiden, Netherlands

**Keywords:** leukocyte migration, metabolism, nontuberculous mycobacterium, Tlr2, zebrafish

## Abstract

*Mycobacterium avium* is the most common nontuberculous mycobacterium (NTM) species causing infectious disease. Here, we characterized a *M. avium* infection model in zebrafish larvae, and compared it to *M. marinum* infection, a model of tuberculosis. *M. avium* bacteria are efficiently phagocytosed and frequently induce granuloma-like structures in zebrafish larvae. Although macrophages can respond to both mycobacterial infections, their migration speed is faster in infections caused by *M. marinum*. Tlr2 is conservatively involved in most aspects of the defense against both mycobacterial infections. However, Tlr2 has a function in the migration speed of macrophages and neutrophils to infection sites with *M. marinum* that is not observed with *M. avium*. Using RNAseq analysis, we found a distinct transcriptome response in cytokine-cytokine receptor interaction for *M. avium* and *M. marinum* infection. In addition, we found differences in gene expression in metabolic pathways, phagosome formation, matrix remodeling, and apoptosis in response to these mycobacterial infections. In conclusion, we characterized a new *M. avium* infection model in zebrafish that can be further used in studying pathological mechanisms for NTM-caused diseases.

## Introduction

The infectious diseases caused by mycobacterial pathogens other than the *Mycobacterium tuberculosis* (Mtb) and *M. leprae* complexes, are collectively called nontuberculous mycobacteria (NTM) infections (1). NTM include approximately 200 species and are ubiquitously distributed in the environment, like soil, dust, and water (2). Currently, NTM infectious diseases have provoked wide attention because of the rise of their incidences globally (3, 4). Although there are existing treatments for some NTM infectious diseases, the treatment regimens are long and have a high frequency of multi-drug resistant cases (5). Thus, it is urgent to discover novel diagnostics and therapeutic strategies for patients infected with NTM. Currently, host-directed therapies (HDT) are one of the most promising strategies to combat NTM infectious diseases by making the NTM antibiotic treatment regimens more effective (6–8). However, the current knowledge of the mechanisms underlying host-NTM bacteria interactions is limited and therefore more studies are urgently needed.

The *M. avium* complex (MAC), which consists of the *M. intracellulare* and *M. avium* species, is one of the most common disease-causing NTM group (9–11). Although MAC bacteria are generally believed to be less virulent for primates than Mtb, they can cause pulmonary and extra-pulmonary disease in susceptible individuals, e.g., patients with acquired immunodeficiency syndrome (AIDS) or with a history of lung disease (12–14). To be noted, Mtb infected patients can be dually infected with MAC bacteria (15). Unfortunately, there are only multipronged treatment approaches for the MAC infections available (15). That is, among other reasons, because developing new drugs or treatment regimens is challenging due to the limited research and sometimes results are contradictory between *in vitro* and mice *in vivo* studies (16, 17), or between studies using different subspecies of *M. avium*. *M. avium* has at least four subspecies, and it has been demonstrated that they cause different disease characteristics (18, 19). Standardized MAC infectious disease animal models are therefore needed to study the mechanism of MAC infection and test new drugs effectively. In previous studies, the *M. avium* Chester (also called MAC 101) infectious capacity has been evaluated in different mouse strains, including BALB/c, C57BL/6, nude, and beige mice, allowing for drug or treatment assessment (20, 21). Thus, MAC 101 can be considered as a standard strain to investigate *M. avium* infection studies.

Zebrafish (*Danio rerio*) larvae are popular as a model to study human infectious disease because their innate immune system is highly similar to that of mammals and they are optically accessible making the infectious agents and immune cells easy to track *in vivo* (22). Furthermore, they enable investigation of innate immune function in isolation from adaptive immunity (22–24). Zebrafish larvae have been an effective model organism to study the mechanism of Mtb infection for over 15 years (25). A majority of the studies have used *M. marinum*, a natural pathogen of fish, as the infectious agent because it is genetically closely related to Mtb, and has been shown to cause granuloma formation in zebrafish larvae at high frequency (26). Recently, zebrafish also was used as an animal model for the investigating the pathogenesis mechanism of some NTM infections, e.g *M. abscessus* (27–29), *M. fortuitum* (30), and *M. kansasii* (31).

Innate immune cells largely depend on pattern recognition receptors (PRRs) to initiate protective innate immune responses in the host against invading pathogens (32). Toll-like receptor 2 (TLR2) serves as one of the most important PRR to sense such invading pathogens through pathogen-associated molecular patterns (PAMPs) (33). Much progress has been made the last decades in revealing the function of TLR2 in defense against Mtb infection. It has been reported that TLR2 senses invading Mtb bacteria through the lipoproteins and glycolipids located on their cell wall (34–36), initiating pro-inflammatory responses promoting bacterial clearance (37). However, it has been shown that activation of TLR2 also activates anti-inflammatory responses (38). The PRR feature of TLR2 makes it popular as a therapeutic target for TB (39). However, there is little known about the involvement of TLR2 in *M. avium* infection.

In this study, we developed an innovative zebrafish larval infectious model for studying *M. avium* infection. Moreover, we compared the innate immune response of zebrafish larvae to infection with two different species of NTM, *M. marinum* Mma20 and *M. avium* MAC 101, specifically with regard to the bacterial burden, electron microscopy, live imaging analysis, and transcriptomic gene expression profiles. Using this system, we analyzed the function of *tlr2* during the infection with both mycobacterial species with special attention to the responsive cell migration behavior.

## Materials and methods

### Zebrafish husbandry

The husbandry of adult zebrafish lines and all zebrafish experiments described in this study was in accordance with guidelines from the local animal welfare committee (DEC) of the university (License number: protocol 14,198), in compliance with the international guidelines specified by the EU Animal Protection Directive 2010/63/EU, and was conducted according to standard protocols (www.zfin.org). There was no adult zebrafish sacrificed in this study. All experiments were done with zebrafish larvae developed within 5 dpf, therefore prior to the free-feeding stage and did not fall under animal experimentation law according to the EU Animal Protection Directive 2010/63/EU. Zebrafish eggs and larvae were cultured and grown at 28.5°C in egg water (60 g/ml Instant Ocean sea salts). Zebrafish larvae were anesthetized with egg water containing 0.02% buffered 3-aminobenzoic acid ethyl ester (Tricaine, Sigma-Aldrich, Netherlands) for bacterial infection and imaging experiments.

The ABTL wild type zebrafish strain, *tlr2^sa19423^* mutant (ENU-mutagenized) and the offspring of its wild type siblings or the following transgenic lines: *Tg (mpeg1:EGFP)^gl22^*, *tlr2^+/+^ Tg (mpeg1:mCherry-F);TgBAC (mpx: EGFP)* and *tlr2^−/−^ Tg (mpeg1:mCherry-F);TgBAC (mpx: EGFP)* were used for this study (38).

### Bacterial strain culture

The *M. marinum* m20 (Mma20), the *M. avium Chester* (also called MAC 101, ATCC^®^ 700898™), Mma20 expressing mCherry fluorescent protein (26), MAC 101 containing the Wasabi expression vector pSMT3 (Addgene, plasmid 26589), and MAC 101 expressing DsRed through pND239 plasmid (40) were used in this study to induce infection in zebrafish embryos. The Mma20 and MAC 101 without any fluorescent protein were grown at 28.5°C in Middlebrook 7H9 broth with acid-albumin-dextrose-catalase (ADC) enrichment or Middlebrook 7H10 agar with 10% oleic acid-albumin-dextrose-catalase (OADC) enrichment. The Mma20 mCherry, MAC 101 Wasabi and MAC 101 DsRed were grown in the same medium or plates with hygromycin 50 µg/mL.

### Alexa Fluor dye staining of mycobacteria

To visualize the interaction between the mycobacteria and leukocytes, the succinimidyl esters (NHS ester) of Alexa Fluor 647 (Invitrogen, A20006) was applied to stain the mycobacteria. The dye was dissolved in high-quality, anhydrous dimethylsulfoxide (DMSO) at a final concentration of 5 mg/mL for preparing the reactive dye solution. For this method, Mma20 and MAC 101 were cultured in 7H9 broth based on the description above and were harvested in the logarithmic phase. The mycobacterial strains were re-suspended in 250 µL 0.1 M sodium bicarbonate buffer (NaHCO_3_, pH 8.3) and then slowly added 10 µL of the reactive dye solution. The mixture was incubated at room temperature for 20 min. Subsequently, the stained mycobacteria were washed twice by sterile PBS. The Alexa Fluor strained Mma20 and MAC 101 were used for the cell tracking and the cell recruitment assay.

### Microinjection

Liquid cultures of Mma20 and MAC 101 were harvested and prepared for the microinjection, according to procedures described before in (41). In short, mycobacterial strains were grown to the logarithmic phase and harvested by centrifugation and washing three times in sterile PBS. Subsequently, bacterial suspensions were re-suspended in sterile PBS with 2% polyvinylpyrrolidone (PVP40) with the desired concentration by measuring the OD600. An OD600 of 1 corresponds to approximately 10^8^ MAC 101, which is the same as Mma20. Embryos were systemically infected with mCherry-labeled Mma20 or Wasabi-labeled MAC 101 through blood island infection at 28 hpf by using the method described in (41). Before we quantified the bacterial burden by the fluorescence, we analyzed the correlation between MAC 101 CFU and average fluorescent signal (**Supplementary Figure 1**). To observe macrophage and neutrophil migration behavior upon mycobacterial infection, zebrafish tailfin infection model was applied (42, 43). For the live imaging, zebrafish larvae were locally infected in the tail fin at 3 dpf with ~50 CFU Mma20 or MAC 101 as previously described (42, 43).

### Imaging and quantification of bacterial burden

Mycobacterial infected ABTL, *tlr2^+/+^*, and *tlr2^-/-^* zebrafish larvae were imaged at 1 dpi and 4 dpi for the quantification of the bacterial burden changes by using a Leica M205FA fluorescence stereomicroscope, equipped with a Leica DFC 345FX camera. All experiments were performed three times independently and in the same microscope setting. The integrated intensity of bacterial loads was quantified by using Quantifish software (https://github.com/DavidStirling/QuantiFish) (44).

### Confocal microscopy imaging

Confocal microscopy imaging was applied for the observation of the granuloma-like cluster and the investigation of the leukocyte migration behavior upon two mycobacterial infections. Observed larvae for each condition were embedded in 1% low melting point agarose (Sigma Aldrich) with 0.02% tricaine and imaged under a Leica TCS SP8 confocal microscope (Leica Microsystems). 4 dpi blood island infected larvae were imaged with a 20× objective (N.A. 0.75) to observe the phenotype of the granuloma-like clusters upon two different mycobacterial infections. In order to investigate the leukocyte migration behavior upon two mycobacterial infections, live imaging was performed on 1 hpi tail fin infected larvae with a 1 min time interval for 2 h imaging using a 20× objective (N.A. 0.75). Acquisition settings for the live imaging were kept the same across the groups.

### Transmission electron microscopy

Mycobacteria in tail fin of 5 dpf zebrafish larvae as previously described (43). Wild type zebrafish larvae were infected with ~250 CFU *M. marinum* or *M. avium* at 2 dpf. At 3 dpi, the infected larvae were fixed in 2% glutaraldehyde and 2% paraformaldehyde in sodium cacodylate buffer (pH 7.2) for 3 h at room temperature after anesthetized properly. Subsequently, fixed samples were kept at 4°C for a further 16 h fixation. The next day, the samples were fixed in 1% osmium tetroxide in sodium cacodylate buffer (with 15mgr Potassium Ferrocyanide/ml) for 1 h at room temperature. All samples were kept in epoxy resin (Agar Scientific, AGR1043) for 16 h after the dehydration through a series of ethanol. Ultrathin sections were collected on Formvar coated 200 mesh or one hole copper grids (Agar Scientific, AGS162) stained with 2% uranyl acetate in 50% ethanol and lead citrate for 10 min each. The samples were imaged on a JEM- JEOL 1400 transmission electron microscope (Tokyo, Japan), which was equipped with an Olympus Megaview camera (Tokyo, Japan).

### RNA isolation, deep sequencing and data analysis

To compare the difference of the larvae infected with *M. marinum* Mma20 infection or *M. avium* MAC 101 infection, fifteen 4 dpi ABTL wild type larvae infected with ~250 CFU Mma20 (four replicates) or ~4500 CFU MAC 101 (four replicates) were collected for the total RNA isolation. The same amount of ABTL wild type larvae (four replicates) were injected with sterile PBS as a control group. The total RNAs were isolated by using TRIzol Reagent (Life Technologies) to create RNAseq libraries. Moreover, DNase treatment (Thermo Fisher Scientific, EN0525) was applied to eliminate the effect of the DNA from the samples following the manufacturer’s instructions. The concentration and quality of RNAs were assessed by NanoDrop 2000 (Thermo Fisher Scientific, the Netherlands).

The deep sequencing was performed in the company GenomeScan (GenomeScan B. V., Plesmanlaan 1d, 2333 BZ, Leiden, Netherlands). The RNAseq libraries were sequenced by applying a NovaSeq 6000 v1.5 device. Image analysis, base calling, and quality check were done by the Illumina data analysis pipeline RTA3.4.4 and Bclfastq v2.20. Subsequently, RNAseq reads were aligned against the zebrafish genome (GRCz11) by using CLC genomic workbench software (QIAGEN, Cat. 832583). The percent of aligned reads mapping is exceeding 90% among all samples in this study. Data is available at NCBI GEO, series record GSE218892.

The PCA for RNAseq tool from CLC genomic workbench was utilized for clustering samples. The Differential Expression in Two Groups tool from the CLC genomic workbench was used to acquire the DEGs between the mycobacterial infection and its control groups. In brief, the tool performs a statistical differential expression test based on a negative binomial Generalized Linear Model (GLM) (See the user manual of the CLC genomic workbench, page 829: https://resources.qiagenbioinformatics.com/manuals/clcgenomicsworkbench/current/User_Manual.pdf) (45). A cut-off setting of the FDR p-value < 0.05 and |FoldChange| > 1.5 was used to define significantly regulated DEGs. The defined significantly regulated DEGs were used for further GO analysis and KEGG pathway enrichment analysis by using DAVID (http://david.abcc.ncifcrf.gov/summary.jsp). The visualization of the PCA plot and KEGG enrichment analysis was performed in R 4.2.1. Pathvisio 3.3.0 (https://pathvisio.github.io/downloads) was applied for the visualization of the significantly regulated genes in the pathways (46).

### Cell tracking and its quantification

The 4D files of leukocyte tracking generated from time-lapse acquisitions were processed by using Imaris x64 7.4 (Bitplane) or ImageJ (NIH, Bethesda, ML, USA). An automatic 3D cell tracking algorithm in Imaris x64 7.4 (Bitplane) was employed to build macrophage or neutrophil trajectories in the live imaging of mycobacterial infected larvae. The data of the number, mean speed, and meandering index of recruited leukocytes in the infected tail fin region were output from the Imaris software.

### Statistical analyses

**Figure 1:**
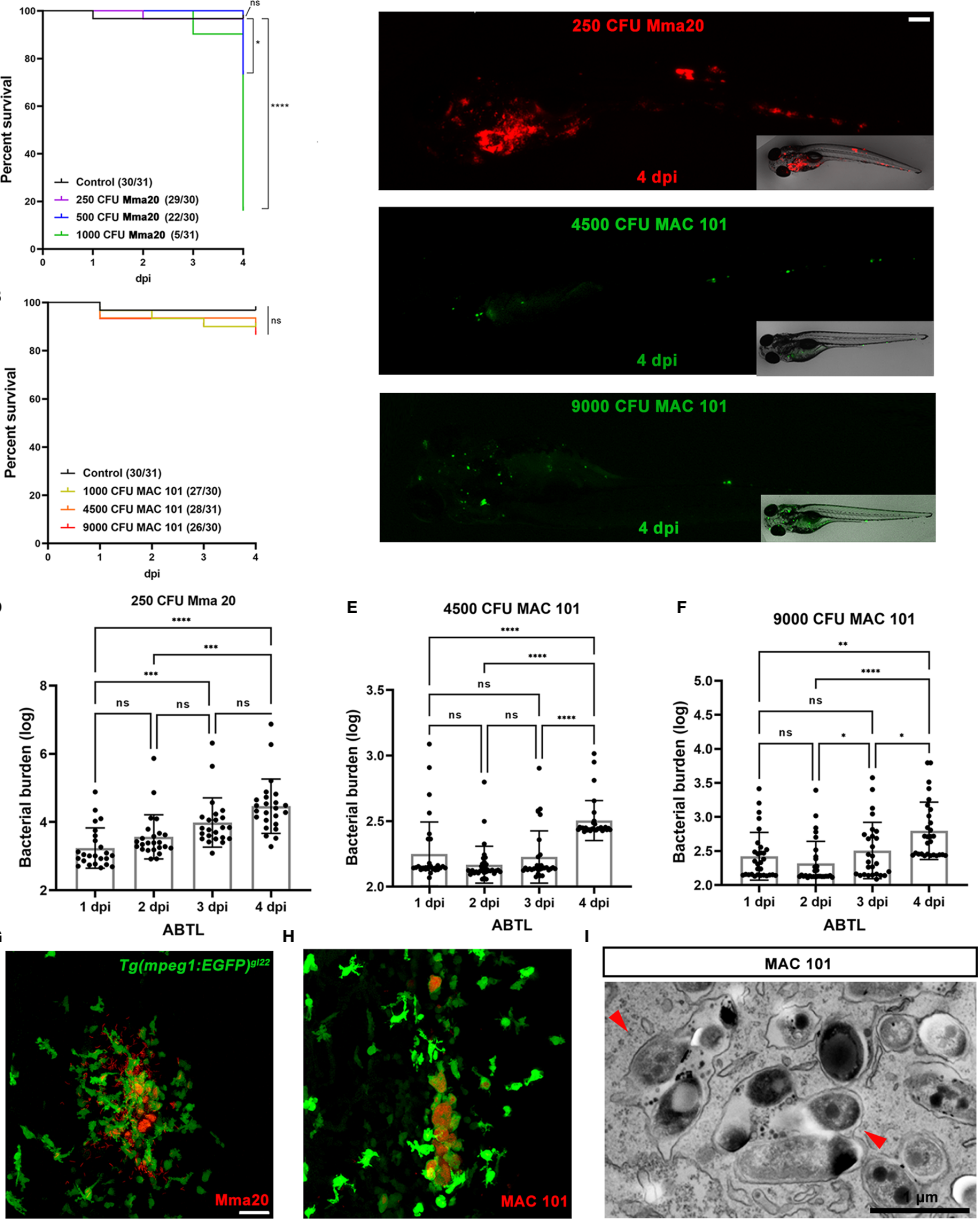
Characterization of *M. avium* infection in zebrafish larvae compared to *M. marinum* infection. **(A, B)** Percent of survival curves for ABTL zebrafish larvae infected with a series of doses *M. marinum* Mma20 or *M. avium* MAC 101. ABTL zebrafish larva infected with mCherry-labeled *M. marinum* Mma20 at a dose of ~250 CFU and infected with wasabi-labeled *M. avium* MAC 101 at a dose of ~4500 CFU or 9000 CFU by caudal vein infection at 28 hpf. **(C)** Representative images for the bacterial burden quantification were taken at 4 dpi. Scale bar: 50 µm. **(D)** Bacterial burden quantification of ABTL zebrafish larvae upon ~250 CFU Mma20 infection. **(E)** Bacterial burden quantification of ABTL zebrafish larvae upon ~4500 CFU MAC 101 infection. **(F)** Bacterial burden quantification of ABTL zebrafish larvae upon ~9000 CFU MAC 101 infection. **(G, H)** Representative CLSM images of *Tg(mpeg1: EGFP)^gl22^* zebrafish larvae infected with mCherry-labeled Mma20 strain **(G)** or DsRed-labeled MAC 101 **(H)**. *Tg(mpeg1: EGFP)^gl22^* embryos were infected ~250 CFU Mma20 mCherry strain or ~4500 CFU MAC 101 DsRed strain at 28 hpf. CLSM images were taken for the 4 dpi infected larvae by using 40 times magnification lens (oil immersion, N.A. 1.3). Scale bar: 50 µm. **(I)** TEM pictures showing a sagittal section through MAC 101 in wild type zebrafish larva. Red arrows represent the bacteria inside of a phagocyte. Scale bar: 1 µm. In **(A, B)** data were collected from three pools of zebrafish larvae. In (D, E, and F) data (mean ± SD) were combined from three pools of zebrafish larvae. Statistical significance of differences was determined by using one-way ANOVA with Tukey’s multiple comparison test as a *post-hoc* test. *P < 0.05, **P < 0.01, ***P < 0.001, ****P < 0.0001. Sample size (n): 24, 24, 23, 24 **(D)**, 31, 33, 31, 30 **(E)**, 30, 29, 27, 29 **(F)**.

**Figure 2:**
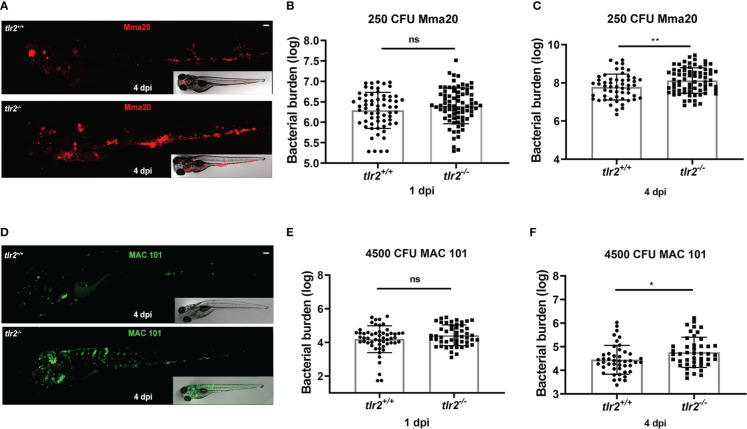
Quantification of bacterial burden in *tlr2* zebrafish larvae with *M. marinum* or *M. avium* infection. *Tlr2^+/+^* and *tlr2^−/−^* embryos were infected at 28 hpf by caudal vein infection with mCherry-labeled *M. marinum* strain Mma20 at a dose of ~ 250 CFU, or infected with ~ 4500 CFU wasabi-labeled *M. avium* strain MAC 101. **(A)** Representative images of *tlr2^+/+^* and *tlr2^−/−^* embryos infected with mCherry-labeled *M. marinum* strain Mma20 at 4 dpi. **(B, C)** Quantification of bacterial burden of *tlr2^+/+^* and *tlr2^−/−^* upon Mma20 infection at 1 dpi and 4 dpi. **(D)** Representative images of *tlr2^+/+^* and *tlr2^−/−^* embryos infected with wasabi-labeled *M. avium* strain MAC 101 at 4 dpi. **(E, F)** The bacterial burden of *tlr2^+/+^* and *tlr2^−/−^* upon MAC 101 infection were quantified at 1 dpi and 4 dpi. In (B, C, and E, F) data (mean ± SD) were combined from three independent experiments. Statistical significance of differences was determined by unpaired t-test for comparison between the *tlr2* mutant and wild type group. ns, non-significant; *P < 0.05, **P < 0.01. Scale bar: 50 µm. Sample size (n): 64, 78 **(B)**, 54, 72 **(C)**, 54, 50 **(E)**, and 45, 45 **(F)**.

**Figure 3:**
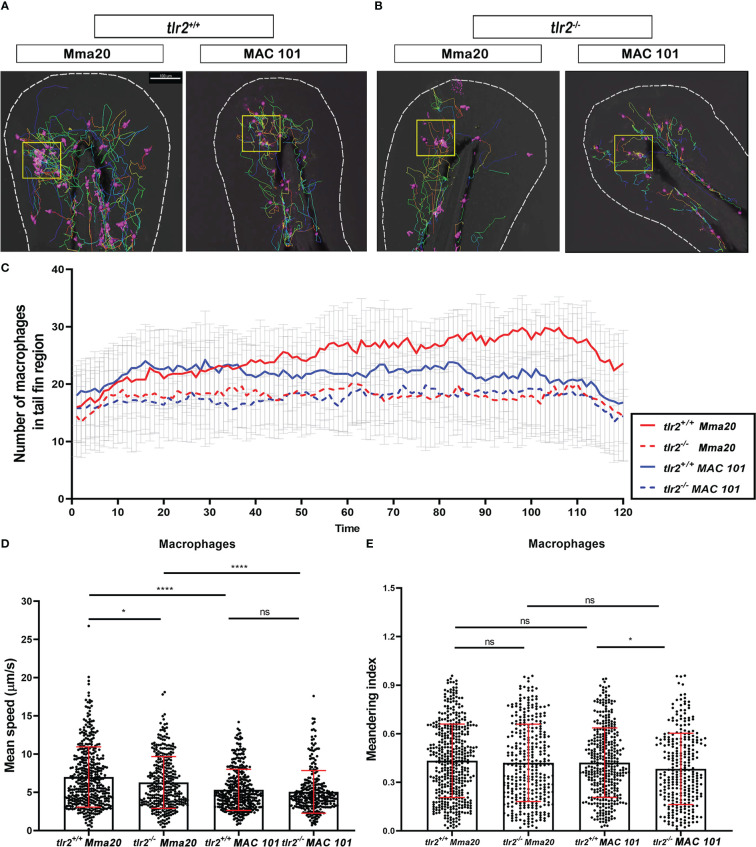
Quantification of macrophages behavior in *tlr2* mutant and wild type control larvae after *M. marinum* Mma20 or *M. avium* MAC 101 tail fin infection. **(A, B)** Representative images of macrophage tracks in *tlr2^+/+^* or *tlr2^-/-^* larvae with Mma20 or MAC 101 tail fin infected. The magenta balls represent the tracked macrophages, the yellow box indicates the infected area. **(C)** The number of recruited macrophages to the tail fin region upon Mma20 or MAC 101 infection. The curves represent the mean value of the recruited macrophage numbers at different time points. **(D)** The mean speed of individual tracked macrophages in the tail fin region. **(E)** The meandering index of tracked macrophages in the tail fin region. In **(D, E)** data (mean ± SD) were combined from three independent experiments with 5 fish in each group. Two-way ANOVA with Tukey’s multiple comparison test as a *post-hoc* test. ns, non-significant; *P < 0.05, ****P < 0.0001. Scale bar: 100 µm; Sample size (n): 447, 343, 372, 290 **(D, E)**.

**Figure 4:**
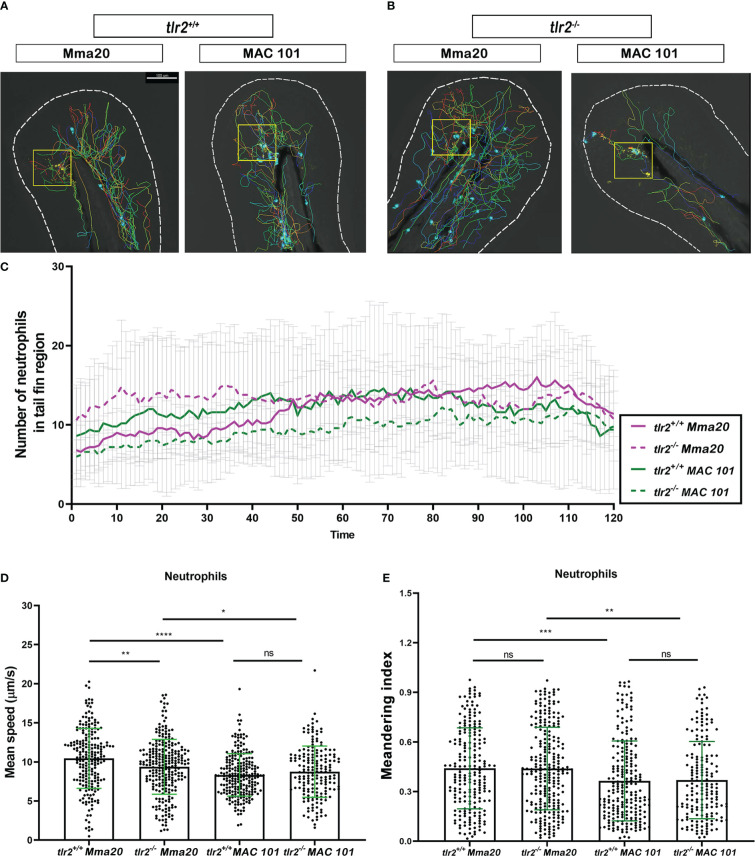
Quantification of neutrophils behavior in *tlr2* mutant and wild type control larvae after *M. marinum* Mma20 or *M. avium* MAC 101 tail fin infection. **(A, B)** Representative images of neutrophil tracks in *tlr2^+/+^* or *tlr2^-/-^* larvae with Mma20 or MAC 101 tail fin infected. The cyan balls represent the tracked neutrophils, the yellow box indicates the infected area. **(C)** The number of recruited neutrophils to the tail fin region upon Mma20 or MAC 101 infection. The curves represent the mean value of the recruited neutrophil numbers at different time points. **(D)** The mean speed of individual tracked neutrophils in the tail fin region. **(E)** The meandering index of tracked neutrophils in the tail fin region. In **(D, E)** data (mean ± SD) were combined from three independent experiments with 5 fish in each group. Two-way ANOVA with Tukey’s multiple comparison test as a *post-hoc* test. ns, non-significant; *P < 0.05, **P < 0.01, ***P < 0.001, ****P < 0.0001. Scale bar: 100 µm; Sample size (n): 217, 254, 228, 179 **(D, E)**.

The statistical analysis of **Figures 1**–**4**, and **Supplementary Figure 2** was done by using Graphpad Prism software (Version 9.0.0; GraphPad Software, San Diego, CA, USA). All experiment data in this study are shown as mean ± SD. D’ Agostino-Pearson omnibus normality test was performed to determine the normal (Guassian) distribution of the data. In **Figures 3D, E** and **Figures 4D, E**, statistical significance of differences was determined by two-way ANOVA with Tukey’s Multiple Comparison test as a *post-hoc* test. The other experiments were analyzed by using unpaired, two-tailed t-tests for comparisons between two groups and one-way ANOVA with Tukey’s multiple comparison methods as a *post-hoc* test for comparisons between more than two groups. Significance was established at *P* < 0.05 and the other significance levels are indicated as * *P* < 0.05; ** *P* < 0.01; *** *P* < 0.001; **** *P* < 0.0001.

## Results

### *M. avium* bacteria are efficiently phagocytosed and induce granuloma like structures in zebrafish larvae

To test the virulence of *M. avium* MAC 101 in a zebrafish model we infected larvae with increasing dosages of bacteria carrying fluorescent protein reporters. As a control we used the established *M. marinum* strain Mma20 infection protocol (41). We infected the larvae systemically by injection into the caudal vein at 28 hours post fertilization (hpf) and monitored larval survival and infectious development by fluorescent microscopy over the following 4 days (**Figure 1**). The results clearly demonstrate that infection with Mma20 is drastically more lethal than with MAC 101 over the 4-day assessment period (**Figures 1A, B**). For example, only ~73% of larvae infected with 500 CFU Mma20 survived until 4 days post infection (dpi) (**Figure 1A**). The results show that even at the highest tested infectious dose of 9000 colony forming units (CFU) of MAC 101, ~87% of larvae survived until the end of the experiment, and with lower dosages of 4500 and 1000 CFU, the survival was higher than 90% (**Figure 1B**). The representative images for the bacterial burden quantification are shown in **Figure 1C**. We subsequently assessed the infectious development of MAC 101 and Mma20 by microscopy-based analysis, using fluorescent signal derived from the injected bacteria as a proxy for the infectious status in the larvae. Larvae infected with 250 CFU Mma20, 4500 CFU, and 9000 CFU MAC 101 all exhibited significant increases in the fluorescent signal at 4 dpi compared with 1 dpi (**Figures 1D** **–F**), indicating a progressing infection despite the low overall mortality in these groups. Interestingly, while the fluorescent signal in Mma20- infected larvae rose steadily from 1 to 4 dpi, MAC 101- infected larvae exhibited a non-significant drop in fluorescent signal from 1 to 2 dpi, only to recover and grow at 3 and 4 dpi. This underscores the different dynamics of infection between these two species of mycobacteria.

In order to investigate the role of macrophages in MAC 101 granuloma formation we used larvae of the Tg(*mpeg1*:EGFP)^gl22^ zebrafish line, in which macrophages express the green fluorescent protein EGFP. In order to compensate for the much faster replication speed and lethality resulting of infection with *M. marinum* compared to infection with *M. avium* bacteria, we used a higher CFU doses for *M. avium*. We found that only the dose of 250 CFU infection had a survival rate higher than 90% in the *M. marinum* infection groups (**Figure 1A**). In the *M. avium* infection groups, survival rate of the dose of 1000 CFU and 4500 CFU was higher than 90% (**Figure 1B**). However, the infection of 1000 CFU *M. avium* cannot be detected by stereo fluorescent microscopy. Therefore, we chose a dose of 4500 CFU of *M. avium* and a dose of 250 CFU of *M. marinum* to test the formation of granuloma-like clusters by confocal microscopy at 4 dpi (**Figures 1G, H**). This close examination revealed that both pathogens efficiently form granuloma-like clusters of macrophages with phagocytosed mycobacteria, but that a few notable differences exist between them. While it is common to find a certain fraction of bacteria not inside macrophages in the case of Mma20 infection (**Figure 1G**), MAC 101 is virtually always found exclusively intracellularly in macrophages (**Figure 1H**). Given that Mma20 is usually phagocytosed completely within 30 minutes of infection (47), this is most likely a reflection of a more rapid progression of the infection with Mma20.

To explore the ultrastructure of granuloma-like clusters after infection with *M. avium*, we performed transmission electron microscopy (TEM). For this purpose, we used the tail fin infection method, which is suited for observing the interaction between host and injected microbes because the tail fin of zebrafish larvae only consists of two epithelial cell layers and normally has no leukocytes infiltrating the tissue (42, 43, 48). For this purpose, ~250 CFU DsRed labeled MAC 101 were injected into the tail fin of Tg(*mpeg1*:EGFP)^gl22^ larvae at 2 days post fertilization (dpf), samples were prepared and TEM images acquired at 3 dpi (**Figure 1I**). The results show that *M. avium* MAC 101 was observed frequently inside phagocytes and surrounded by a phagosomal membrane (**Figure 1I**).

In conclusion, *M. avium* infection in zebrafish larvae leads to the efficient phagocytosis and formation of granuloma like structures. Thereby it is a good model to further study the function of macrophage host defence against clinically relevant NTM bacteria and to compare it with tuberculosis studies.

### The protective role of Tlr2 is conserved in *M. marinum* and *M. avium* infection

Previous studies by our group have demonstrated that *tlr2* is important for the ability of zebrafish larvae to control *M. marinum* infection, a phenomenon which can be explained by the effect on metabolic pathways and the presence of higher extracellular bacterial burden in the *tlr2* mutants (49). To further explore the role of *tlr2* in the control mycobacterial infection, we investigated whether *tlr2* is also involved in the immune response to *M. avium* infection. We therefore injected MAC101 bacteria into *tlr2* loss-of-function mutants (*tlr2* ^sa19423/sa19423^) and their wild type siblings (*tlr2^+/+^*). We infected the larvae in the same manner as before, with ~250 CFU *M. marinum* Mma20 or ~4500 CFU *M. avium* MAC 101 respectively. Images of infected larvae were taken at 1 dpi and 4 dpi to assess the bacterial burden by integrated intensity. In the representative images of the *tlr2* zebrafish larvae upon *M. marinum* infection, we found more and bigger granuloma-like clusters in the *tlr2^-/-^* upon *M. marinum* infection (**Figure 2A**). Although there was no significant difference between *tlr2^+/+^* and *tlr2^-/-^* larvae upon *M. marinum* infection at 1 dpi, the bacterial burden was significantly increased in the *tlr2^-/-^* group at 4 dpi (**Figures 2B, C**). These results are consistent with our previous study (49). In the *M. avium* infection groups, we also found that the bacterial burden was significantly increased in *tlr2^-/-^* zebrafish at 4 dpi and no difference was found at 1 dpi (**Figures 2D** **–F**). In conclusion, *tlr2* plays a protective role in infection with *M. avium* and *M. marinum*. Interestingly, the distribution of the *M. avium* burden in *tlr2^-/-^* was likely intravascular while the distribution of *M. marinum* in *tlr2^-/-^* was extravascular (**Figures 2A, D**) indicating a differential role of TLR2 in responses to these different bacteria.

### Migration speed of macrophages and neutrophils towards infecting *M. avium* is slower than towards *M. marinum*

Previous studies have demonstrated that the migration of leukocytes during the infection process is important for bacterial clearance, containment, dissemination, and granuloma formation at the early mycobacterial infectious stage (28, 43, 50, 51). To study the recruitment of macrophages and neutrophils to the sites of the infection, we used the tail fin infection model in both wild type and *tlr2* mutants. The thin tail fin makes it possible to set short time interval when the cell tracking was performed by CLSM, enabling high accuracy of tracing individual cells.

For using this method, 50 CFU of Alexa Fluor dye stained Mma20 or MAC 101 bacteria were injected into 3 dpf *tlr2^+/+^* Tg(*mpeg1.1:*mCherryF*^ump2^*; *mpx:*GFP^i113^). Time-lapse microscopy was performed by using confocal laser scanning microscopy (CLSM) between 1 hour post infection (hpi) to 3 hpi. Representative images and trajectories of macrophages and neutrophils are shown in **Figures 3A**, **4A**, respectively. The time-lapse images were analyzed by Imaris software to quantify the number, the speed of migration and meandering index of recruited leukocytes to the infection area in the tail fin region (**Figures 3**, **4**). The data shows a trend of more macrophages recruited to the *M. marinum* than to the *M. avium* bacteria although at individual time point there is no statistical significance (**Figure 3C**). We further quantified the speed and meandering index of the macrophages in the tail region. As shown in **Figure 3D**, there is significant difference in migration speed of macrophages towards the *M. avium* and *M. marinum* infection sites (*P* < 0.0001). However, the meandering index of the macrophages is not significantly different in these infection experiments (**Figure 3E**).

We also measured the migration dynamics of neutrophils to the infection sites using the Tg(*mpx*:GFP)^i113^ line. The results show that also neutrophils have a lower mean speed towards the infection site of *M. avium* than *M. marinum* (*P* < 0.0001) (**Figure 4D**). In addition, the meandering index of the neutrophils is also lower in the response to *M. avium* than to *M. marinum* (*P* < 0.001) (**Figure 4E**).

In conclusion there is a considerable difference in the dynamic responses of macrophages and neutrophil towards infection by *M. avium* and *M. marinum* bacteria. As such difference in dynamic responses towards different mycobacterial species has never been reported before, we studied the host genetic basis for this phenomenon in more detail using our *tlr2* mutant zebrafish.

### *Tlr2* differentially controls macrophage and neutrophil migration dynamics after different mycobacterial infections

We have previously shown that *tlr2* is involved in regulating leukocyte migration in response to wound signaling (38). We hypothesized that *tlr2* could also be involved in the regulation of migratory behavior of macrophages and neutrophils to the sites of mycobacterial infection. To test this hypothesis, we performed the same dynamic analysis of leukocyte migration as described above using the mutant zebrafish strain *tlr2* ^sa19423/sa19423^ Tg(*mpeg1.1:*mCherryF^ump2^; *mpx*:GFP^i113^) larvae.

We found that the recruited macrophages were fewer in numbers in the *tlr2* mutants upon Mma20 and MAC 101 infection compared with wild type controls (**Figures 3A** **–C**). We further quantified the speed and meandering index of the macrophages in the tail region (**Figures 3D** **, E**). In the Mma20 infection group, macrophages in *tlr2^-/-^* moved significantly slower than the macrophages in the wild type control group (**Figure 3D**). These results show that tlr2 is not only involved in macrophage migration dynamics in wound signaling but also in response to infection. However, no significant difference was found in the speed of *tlr2^+/+^* macrophages and *tlr2^-/-^* macrophages after MAC 101 infection (**Figure 3D**). Apparently, the function in macrophages mean speed towards infection is highly specific for the triggering factors. Surprisingly the meandering index of macrophages, although not different after infection with *M. marinum*, was decreased after MAC 101 infection (**Figure 3E**) showing a presently still non-understood complexity of macrophage migration towards different infection sources.

Comparing the behavior of neutrophils, we found that fewer neutrophils were recruited to the infected tail fin in the Mma20- infected *tlr2^+/+^* group compared to the *tlr2^-/-^* group at early time points (**Figures 4A** **–C**). In contrast, higher numbers of neutrophils were recruited in *tlr2^+/+^* compared to the *tlr2^-/-^* MAC101 infection group although the difference in the number of neutrophils becomes smaller in the later stage of the tracking among the four groups (**Figure 4C**). We found that the mean speed and meandering index in the *tlr2^-/-^* neutrophils were decreased after Mma20 infection (**Figures 4D, E**). However, no difference in mean speed and the meandering index was observed between the *tlr2* mutant and its wild type controls after MAC 101 infection (**Figures 4D, E**). We found no difference in recruited leukocyte numbers, mean speed and meandering index of macrophages and neutrophils between *tlr2^+/+^* and *tlr2^-/-^* larvae after PBS mock injection, which demonstrates that the differences observed above are dependent on the infection by mycobacteria, and not the damage of the injection (**Supplementary Figure 2**). In conclusion, the results show that *tlr2* differentially regulates the macrophages and neutrophils dynamic behavior after different mycobacterial infections.

### Differential transcriptome responses to *M. marinum* and *M. avium* infection in zebrafish larvae

**Figure 5:**
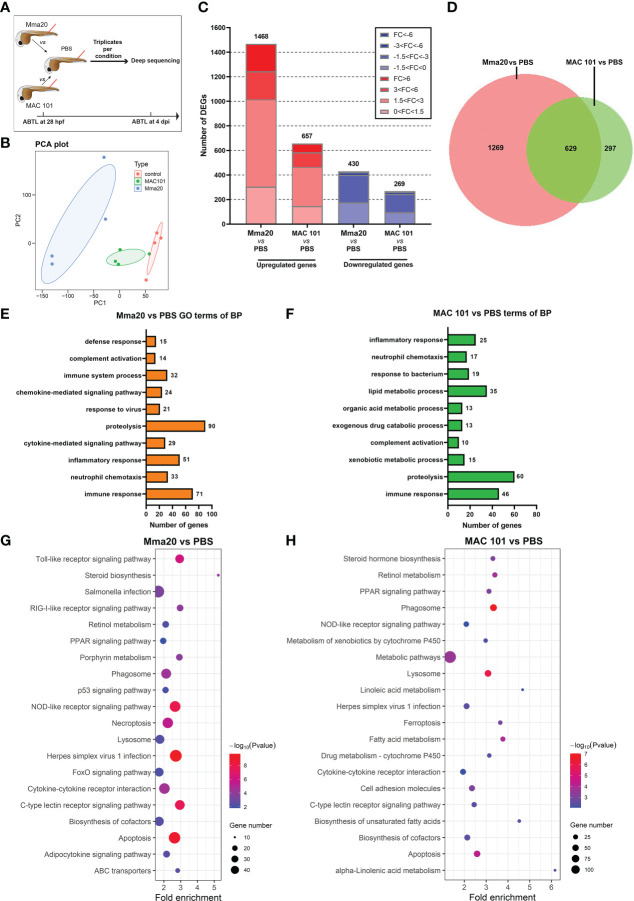
Global transcriptome analysis of *M. marinum* Mma20 vs PBS and *M. avium* MAC 101 vs PBS groups. **(A)** Experimental scheme to collect samples for RNA deep sequencing. AB/TL zebrafish embryos were injected with ~250 CFU *M. marinum* Mma20 strain or ~4500 CFU *M. avium* MAC 101 strain at 28 hpf. The embryos in the control group were injected with sterile PBS with 2% PVP. The samples for RNAseq are taken from four independent sample sets with the injected larvae at 4 dpi. **(B)** Principal component analysis. The RNAseq samples clustered based on their groups, as showed by the solid ellipses grouping samples. **(C)** Overview of the distribution of DEGs fold change in zebrafish larvae infected with Mma20 or MAC 101. DEGs were assessed by FDR p-value < 0.05. Upregulated gene sets are shown in red and downregulated gene sets are shown in blue. The intensity of the color represents the fold change level. **(D)** Venn diagram shows the common and specific DEGs number between the 250 CFU Mma20- infected group and 4500 CFU MAC 101- infected group compared to the control. The Venn diagrams were made by the website: https://www.biovenn.nl/. **(E)** The top 10 gene ontology (GO) terms of biological process with lowest P value in the Mma20- infected larvae compared to the control group. **(F)** The top 10 GO terms of biological process with lowest P value in the MAC101-infected larvae compared to the control group. **(G)** Top 20 significantly enriched KEGG pathways in the Mma20- infected larvae compared to the control group. **(H)** Top 20 significantly enriched KEGG pathways in the MAC 101- infected larvae compared to the control group. The GO analysis and KEGG pathway enrichment analysis were performed by using DAVID. In **(G, H)**, the size of circle represents the enriched gene numbers, bigger circle indicates more enriched genes were found in this pathway. The color of circle represents -log10 (P value).

To gain a better understanding of the differences in leukocyte responses to Mma20 versus MAC 101 we performed transcriptome analysis of zebrafish larvae infected with Mma20 and MAC 101 by RNAseq (**Figure 5**). Mma20 or MAC 101- infected and PBS-injected control groups were collected for RNA isolation at 4 dpi and used to create RNAseq libraries (**Figure 5A**). We used the same doses of bacteria as used for analysis of infection burdens in the *tlr2* mutants (**Figure 2**). Principal component analysis (PCA) showed clear differences between Mma20- infected larvae, MAC 101- infected larvae and PBS- injected controls (**Figure 5B**). When comparing RNAseq data of 4 dpi Mma20 infection larvae to that of PBS-injected control group, we found 1468 genes upregulated and 430 genes downregulated (**Figure 5C**). A different response was observed when larvae were challenged with *M. avium* MAC 101, which exhibited 657 upregulated genes and 269 downregulated genes (**Figure 5C**). To investigate the overlap of genes regulated by the two mycobacteria, we plotted a Venn diagram (**Figure 5D**). The results showed that 629 genes (33.1% in Mma20 *vs* PBS group, 67.9% in MAC 101 *vs* PBS group) were regulated by both Mma20 and MAC 101.We used false discovery rate (FDR) adjusted p-value < 0.05 and |Fold Change| > 1.5 as a significant cutoff for further analysis, we first performed gene ontology (GO) analysis (biological process) by using the online functional classification tool Database for Annotation, Visualization and Integrated Discovery (DAVID; http://david.ncifcrf.gov/summary.jsp) (**Figures 5E, F**; **Supplementary Tables 1, 2**) (52, 53).

The GO analysis of the differential expressed genes (DEGs) comprising the response to the two pathogens revealed many of the same terms, but with some telling differences. The top GO-term in both cases was immune response (**Figures 5E, F**). The rest of the top ten of MAC 101 associated GO terms was dominated by metabolic processes while the top ten Mma20 response was dominated by further inflammatory GO-terms such as inflammatory responses, chemokine and cytokine responses and neutrophil migration (**Figures 5E, F** and **Supplementary Tables 1, 2**). This seems to indicate that the nature of the immune response to MAC101 and Mma20 is either fundamentally different, or it is at a different stage along the same trajectory at this time point.

Subsequently, we classified the differential regulated genes according to KEGG pathways by using DAVID (**Figures 5G, H**). Top 20 significantly enriched KEGG pathways in the Mma20- infected group are shown in **Figure 5G** and **Supplementary Table 3**. Top 20 significantly enriched KEGG pathways in the MAC101- infected groups are shown in **Figure 5H** and **Supplementary Table 4**. There are 25 significantly enriched KEGG pathways found in the Mma20- infected group and 31 significantly enriched KEGG pathways found in the MAC 101- infected group (**Supplementary Tables 3, 4**). Notably, most of the significantly enriched KEGG pathways (21/31) in the MAC 101- infected group are metabolism related pathways (**Supplementary Table 4**), while only 8 pathways (8/25) related to metabolic processes were found in the Mma20- infected group (**Supplementary Table 3**).

**Figure 6:**
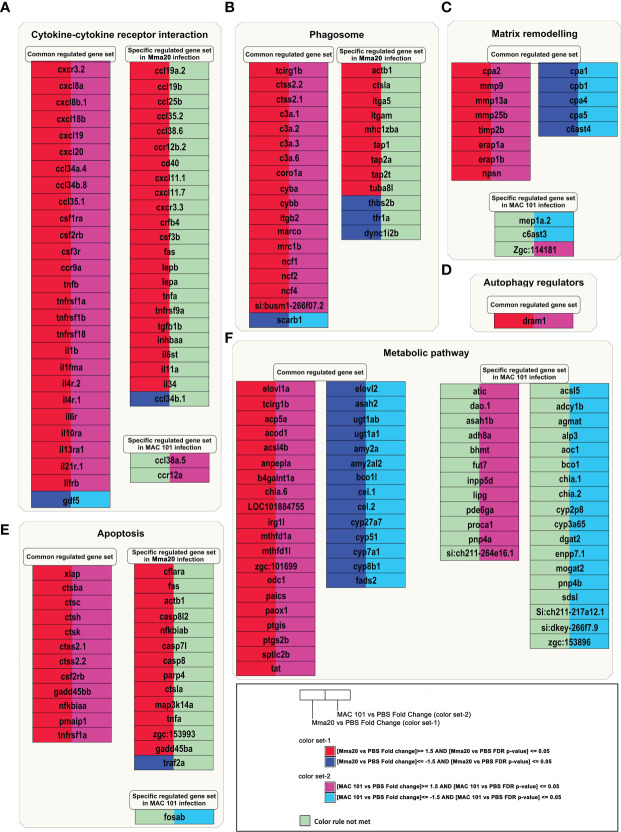
Common and specific gene expression profiles of larvae infected with *M. marinum* Mma20 or *M. avium* MAC 101. DEGs from the Mma20- infected and the MAC 101- infected groups compared to the control group are showing in **(A)** Cytokine and cytokine receptor interaction; **(B)** Phagosome; **(C)** Matrix remodeling; **(D)** Autophagy regulator; **(E)** Apoptosis; **(F)** Metabolic pathway; In the visualization, the gene expression in the comparison of the Mma20- infected and the MAC 101- infected compared to the control group are depicted by color (red, upregulated genes in Mma20- infected group; blue, downregulated genes in Mma20- infected group; Magenta, upregulated genes in MAC 101- infected group; Cyan, downregulated genes in MAC 101- infected group. FDR p-value < 0.05).

We further investigated the common and specific gene expression profiles in *M. marinum* and *M. avium-* infected zebrafish larvae (**Figure 6**). Using FDR adjusted p-value < 0.05 and |Fold Change| > 1.5 as a significance cutoff, we summarized the DEGs in the categories of Cytokine-cytokine receptor interaction (**Figure 6A**); Phagosome formation (**Figure 6B**); Matrix remodeling (**Figure 6C**); Autophagy regulators (**Figure 6D**); Apoptosis (**Figure 6E**) and Metabolic pathways (**Figure 6F**) by using the program Pathvisio (46). In each category, we summarized common regulated gene set, specific regulated gene set in the Mma20- infected group, or specific regulated gene set in the MAC 101- infected group. In the categories of the Cytokine-cytokine receptor interaction (**Figure 6A**), Phagosome formation (**Figure 6B**), and Apoptosis (**Figure 6C**), we show that more specific regulated genes are present in the Mma20- infected groups. However, in some cases the responses of these genes to MAC101 infection are also high but did not reach the threshold of the FDR adjusted p-value. For instance, tnfa was induced 6.1 fold with a FDR adjusted p-value of 0.06. In contrast, more specific regulated genes were found in the categories of Matrix remodeling (**Figure 6C**) and Metabolic pathway (**Figure 6F**) in the MAC 101- infected group. Notably, in the Metabolic pathway, 12 genes were specifically up regulated and 18 genes were specifically down regulated in the MAC 101- infected group (**Figure 6F**).

Overall, the transcriptomic profile of genes characterized as functioning in inflammatory responses and metabolic responses showed divergences in all functional classes of genes, but particularly in Cytokine-cytokine receptor interaction and genes involved in Metabolic pathways. This further underscores that the host immune response to Mma20 and MAC 101 bacteria is very different.

## Discussion

Bacteria belonging to the *M. avium* complex (MAC) are the most important pathogens for NTM infectious disease, accounting for 80% of pulmonary NTM infectious disease cases (54). However, our understanding of the MAC infection mechanism is incomplete. *M. marinum* is a genetically close related to the *M. tuberculosis* complex that is widely utilized to model human tuberculosis *in vivo* (55). Many new insights have been obtained in the last decades in our understanding of tuberculosis disease progression by using *M. marinum* infection in a zebrafish model (24, 25). In this study, we applied the zebrafish larvae to set up a *M. avium* infectious model and characterized the model through the comparison with the *M. marinum* infectious model in three different ways: (I) we compared the difference between the *M. marinum* Mma20 and *M. avium* MAC 101 infection progression microscopically, (II) we focused on the function of Toll-like receptor 2 signaling after Mma20 and MAC 101 infection and (III) we analyzed the common and specific gene expression profiles resulting from the two infections systems using RNAseq. Comparing *M. avium* infection with *M. marinum* infection at these levels could help us to get insights into the mechanisms underlying MAC infectious diseases.

### *M. marinum* Mma20 is more virulent than *M. avium* MAC 101

Injecting fluorescently labeled bacteria allowed us to compare the infectious development of Mma20 and MAC 101. We found that although the bacterial burdens caused by injection of the two tested mycobacterial strains were increasing at 4 dpi (**Figures 1D** **–F**), the pathological progression of *M. avium* infections is different from *M. marinum* infection. Survival rate experiments showed that *M. marinum* infection is more lethal at earlier time points than *M. avium* infection (**Figures 1A, B**). Though both pathogens induce granuloma-like clusters in the host, the morphology of these granuloma-like cluster is different between *M. marinum* infected and *M. avium* infected zebrafish larvae (**Figures 1G, H**). NTM are intracellular pathogens and macrophages are the first responders to defend against NTM at the early infection stage (56). In this study, we found that MAC 101 is persisting inside macrophages with no observable extracellular cording (**Figures 1H, I**). Extracellular cording is a morphology of mycobacteria accompanied by necrotic macrophages and extracellularly replicating bacteria which prevent phagocytosis because of the size of the clusters (57, 58). Bacterial cording is a pathogenic feature associated with hyper-virulence in *M. tuberculosis*, *M. marinum*, *M. abscessus*, *M. fortuitum*, and *M. chelonae* (27, 30, 57, 59, 60).

Macrophages execute a series of functions including recognizing mycobacteria, forming granulomas, and eliminating bacteria (56). However, mycobacteria have evolved the ability to evade the immune system by using macrophages as a safe haven (61, 62). This safe haven in the form of a granuloma affects drug delivery into the mycobacteria inside it, possibly resulting in drug-tolerance (5). Moreover, granulomas can also provide a favorable environment for mycobacteria to survive longer inside the host (63). Thus, the seemingly exclusive presence of MAC 101 bacteria in macrophages infection might contribute to a slow disease progression in MAC infectious diseases and difficulties in treatment. The MAC 101 zebrafish infection model is therefore suitable to be further applied to study the host-mediated mechanism of drug tolerance in macrophages.

### Tlr2 plays a distinct role in defense against different mycobacterial species

In the present study, we found that *tlr2^-/-^* zebrafish larvae showed a higher bacterial burden compared to their wild type controls after either *M. marinum* Mma20 or *M. avium* MAC 101 infection (**Figure 2**). The results with Mma20 are consistent with what we have shown in our previous study with Mma20 infection in *tlr2^-/-^* zebrafish larvae (49). Moreover, it has been demonstrated that mice deficient in TLR2 show increased susceptibility to *M. tuberculosis* infection (64, 65). In agreement, Feng et al. reported that *Tlr2^-/-^* mice showed increased susceptibility to *M. avium* infection compared with their wild type counterparts (66). In contrast, no pronounced difference was observed in *M. avium*-infected *Tlr4^-/-^* mice and infected C57BL/6 mice (66). TLR2 plays a role in active macrophages by recognition of *M. avium* biofilms on their surface (36, 67). Sweet et al. showed that TLR2, but not TLR4, activate macrophages through the interaction with glycopeptidolipids (GPLs) expressed on the surface of *M. avium*, that are related to biofilm formation (67). The study suggests that TLR2, but not TLR4, plays a crucial role in *M. avium* recognition and defense through macrophage activation. However, TLR2 is not the only member from the Toll-like receptor family that can respond to *M. avium* infection and trigger an immune response. TLR6 and TLR9 have also been shown to be required to effectively control *M. avium* growth in mice (68, 69).

Although *tlr2* plays a protective role in the host defense against different NTM species, the underlying mechanisms may be different in infections by different mycobacterial species. The antimicrobial function of TLR2 in macrophages has been previously demonstrated. In both mouse and human macrophages, the clearance of intracellular *M. tuberculosis* bacteria is dependent on TLR2 activation, although the mechanism of the antimicrobial activity is distinct between mouse and human macrophages (70). In mouse macrophages, direct antimicrobial activity triggered by TLR2 is nitric oxide-dependent, however, this process is nitric oxide-independent in human macrophages (70, 71). Liu et al. reported that human macrophage activation by TLR2 is related to vitamin D levels, which sustains the production of the antimicrobial peptide cathelicidin and subsequently leads to killing of intracellular tubercle bacilli (71). In accordance, genes associated with the vitamin D receptor pathway are upregulated in wild type zebrafish larvae while they are downregulated in *tlr2* mutant after *M. marinum* infection (49). This suggests that the higher susceptibility of the *tlr2* mutant to *M. marinum* and *M. avium* infection may be caused by impaired antimicrobial capacity of macrophages.

Different mechanisms of *tlr2*-mediated defense against different mycobacterial species are apparently manifested in different effects on leukocyte behavior. In this study, we found that both macrophages and neutrophils moved faster in *tlr2* wild type larvae than in *tlr2* mutants after Mma20 infection (**Figures 3**, **4**), while *tlr2* deficiency did not affect neutrophil migration in MAC 101 infection (**Figure 4**). The meandering index of *tlr2* mutant macrophages was lower than the *tlr2* wild type macrophages in MAC 101 infection (**Figure 3E**).

We also show that there is a considerable difference in the dynamic responses of macrophages and neutrophils towards infection by *M. avium* and *M. marinum* bacteria independent from *tlr2*. It could be considered that the differences in dynamics of macrophages and neutrophils in migration towards the infection site of *M.marinum* and *M.avium* is caused by the difference in proliferation speed of these two bacterial species. However, it is not expected that a larger number of infected bacteria in the tail fin would lead to different migration dynamics based on the previous findings of Hosseini et al. (43). In that paper it was shown that in the tail fin injection model, *M.marinum* bacteria hardly replicate in the first day after injection. Therefore we don’t expect that in the migration assay the number of bacteria is the factor that explains the different migration dynamics. Since this difference is independent from *tlr2*, we can speculate that the other toll-like receptors are involved in specific recognition of *M. avium*. For example, a good candidate is *tlr8* which we previously have shown to be involved in response to *M. marinum* infection (49). We previously reported that the expression levels of *cxcl11aa* and *cxcll11ac* after Mm infection was higher in 4 dpi *tlr2^+/-^* compared to 4 dpi *tlr2^-/-^* mutant zebrafish larvae (49). Therefore, this altered leukocyte behavior suggests that chemokine expression profiles may be different in *tlr2* mutant zebrafish after infection by different mycobacterial species. Currently, we are performing experiments using organoid culture system with mammalian cells to further study the function of *tlr2* in macrophage migration.

### Common and different transcriptome responses to infection by *M. marinum* and *M. avium*

4To obtain more explanations for the differences we found between *M. marinum* and *M. avium* infection progression in zebrafish larvae, we conducted a deep RNA sequencing to study the whole transcriptome profiles. The used a much higher number of injected bacteria of the *M. avium* strain than the *M. marinum* strain to compensate for the difference in growth speed inside the larvae. The GO enrichment analysis of **Figures 5E**, F showed that the GO term proteolysis (biological process) had the highest DEG numbers in both Mma20 and MAC 101 infection groups compared to the controls (**Figures 5E, F**). Similar results were obtained in a Mtb infected murine bone marrow-derived macrophages study showing an increased proteolytic activity compared to uninfected macrophages (72). Furthermore, cathepsin D and other cathepsins, which are involved in proteolysis, are significantly up regulated in macrophages in human TB granulomas (73). In our study, we also found that these genes are upregulated in both the MAC101 and Mma20 infection groups. It has been reported that abnormal proteolytic activity is associated with wasting syndrome and that *M. avium subsp. paratuberculosis* (MAP) leads to a fatal wasting syndrome in ruminants (74, 75). However, there is no indication that the wasting syndrome observed in *M. avium* infected patients or ruminants is caused by proteolysis dysfunction.

Through KEGG enrichment analysis, we found DEGs are significantly enriched in the cytokine-cytokine receptor interaction pathway in both *M. marinum* and *M. avium* infected zebrafish larvae (**Figures 5G, H**). However, we found that the cytokine-cytokine receptor interaction gene expression profiles are different between the larvae infected with Mma20 and MAC 101 at 4 dpi (**Figure 6A**). *M. marinum* infection induced a higher number of significantly regulated genes from the category of cytokine-cytokine receptor interaction, while there are only two genes, *ccl38a.5* and *ccr12a*, which are specifically up regulated in MAC 101 infection group. *Ccl38*.a is also named *ccl2* (see the website: https://zfin.org), which is chemokine that functions in macrophage migration (76, 77). Since induction levels of these genes is higher with *M.avium* than with *M.marinum* infection, the difference in replication speeds of these bacterial species in the zebrafish larvae does not seem a likely explanation for the different responses. Gene expression analysis in this study suggests that the difference in leukocyte migratory activity in infections with Mma20 and MAC 101 at early time points may be related to various cytokine gene expression patterns. In addition, *M. marinum* infection results in a group of DEGs enriched in the KEGG categories of Phagosome formation and Apoptosis, which correlate with the higher bacterial burdens that were observed in the Mma20- infected larvae (**Figures 1D, G** and **Figures 6B, E**).

Surprisingly, our data showed that *M. avium* infection has a considerably more impact on metabolic processes than *M. marinum* does on zebrafish larvae (**Figures 5E, F**, **Figure 6F**). For example, DEGs in the Mma20- infected groups are only significantly enriched in lipid metabolic process (**Figure 5E** and **Supplementary Table 1**). In contrast, many DEGs in the MAC 101- infected group are enriched in GO terms related to lipid metabolism, including lipid metabolic process, lipid transport, and lipid biosynthetic process (**Figure 5F** and **Supplementary Table 2**). Our results are very comparable to a previous study of a MAP infection in bovine small intestine (78). Through GO analysis, this previous study reported a group of DEGs enriched in lipid storage, lipid metabolic process, and regulation of lipid transport in the subclinical phase of MAP- infected cows (78). MAP- infected macrophages from cows have been shown to accumulate lipid droplets that serve as the carbohydrate energy source for the bacteria (79). Furthermore, intracellular antimicrobial capability has been demonstrated to be impaired in lipid-loaded macrophages upon mycobacterial infection (e.g. Mtb, *M. avium*, and BCG) (6, 80, 81). Therefore, it is interesting to investigate whether aberrant lipid accumulation in macrophages is causing differences of the pathogenic characteristics of *M. avium* infection compared to other mycobacteria.

Besides, we also found several significantly enriched pathways related to metabolism in MAC 101 infection group are similar to the previous study in the MAP- infected cows (78, 82), such as Linoleic acid metabolism, Retinol metabolism, and Arachidonic acid metabolism pathways (**Figure 5H** and **Supplementary Table 4**) (78). In addition, Retinol metabolism, PPAR signaling pathway, Linoleic acid metabolism, Arachidonic acid metabolism, and Arginine and proline metabolism have been shown to be significantly enriched pathways in the ileocecal valve of Holstein cattle with subclinical MAP infection (82), which is similar to our findings in zebrafish (**Figure 5H** and **Supplementary Table 4**).

In conclusion, our findings indicate that the host response to MAC 101 infection involves changes in metabolic processes that are specific to this species. Several mycobacterial infectious diseases, such as tuberculosis, have drawn attention to the significance of immunometabolism and particularly the function of lipids metabolism (83–85). Therefore, it is highly interesting to study the mechanism by which *M. avium* specifically impacts host metabolism.

## Data availability statement

The data presented in the study are deposited in the GEO repository, accession number GSE218892.

## Ethics statement

Larvae for experiments were obtained from zebrafish that were handled in compliance with the local animal welfare regulations and maintained according to standard protocols (zfin.org). The breeding of adult fish was approved by the local animal welfare committee (DEC) of the University of Leiden. No adult animals were used for experimentation. All protocols adhered to the international guidelines specified by the EU Animal Protection Directive 2010/63/EU for which larvae under the age of 5 days post fertilization are not considered test animals.

## Author contributions

WH performed the most biological experiments and wrote the first version of the manuscript, BK performed the mycobacteria infection experiment and assisted with manuscript writing, GL performed the transmission electron microscopy experiment and assisted with manuscript writing, GF-C assisted with manuscript writing, HS supervised the study and initiated the study and has the final responsibility of the manuscript. All authors delivered input for the final version of the manuscript and agreed with its contents.
