# Supplementary material for: Specificity of the innate immune responses to different classes of non-tuberculous mycobacteria
Source: Front Immunol. 2023 Jan 18;13:1075473. doi: 10.3389/fimmu.2022.1075473 (PMC9890051; doi:10.3389/fimmu.2022.1075473)
Supplement: Supplementary file 1 [file DataSheet_1.docx]

Supplementary Material

**
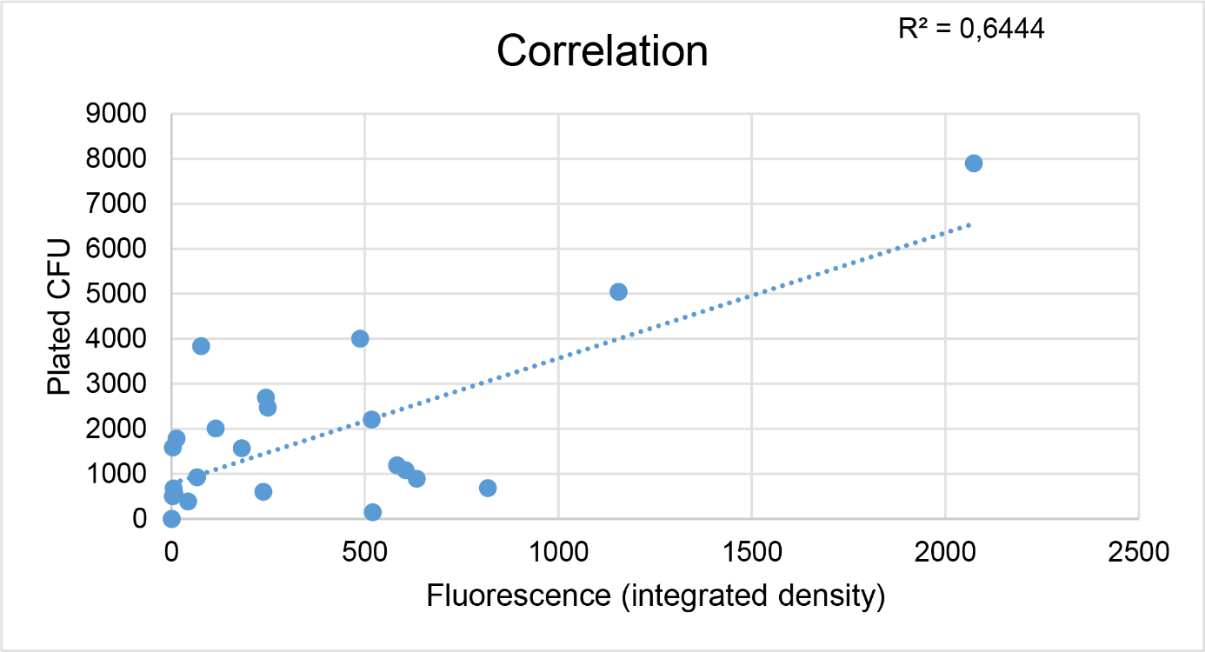
**

**Supplementary Figure 1. The correlation between MAC 101 CFU and average fluorescence signal.**


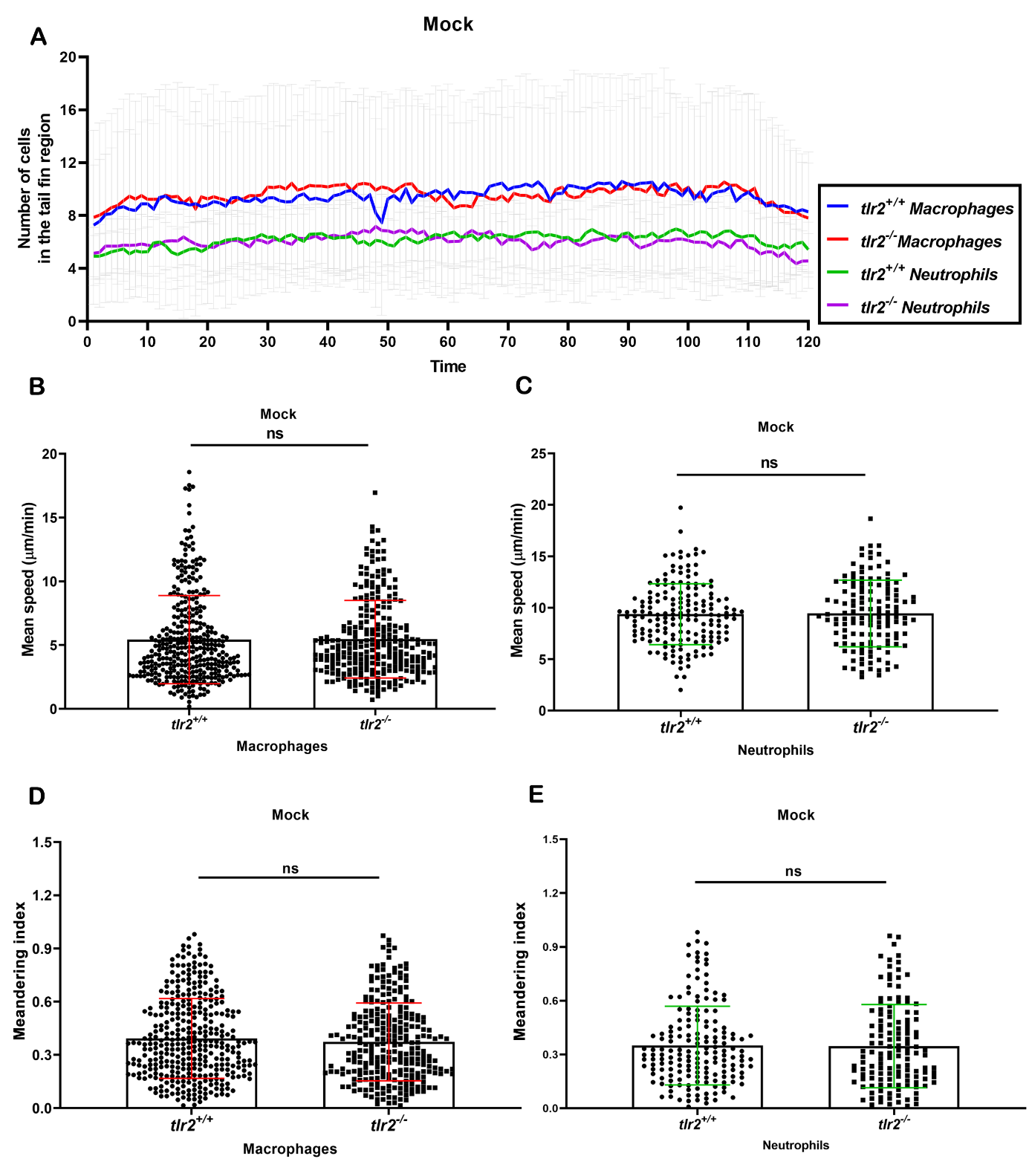


**Supplementary Figure 2. Quantification of leukocytes behavior in *tlr2* mutant and wild type control larvae after mock injection in tail fin.**(A) The number of recruited leukocytes to the tail fin region upon mock infection.
(B) The mean speed of tracked macrophages in PBS injected tail fin region.
(C) The mean speed of tracked neutrophils in PBS injected tail fin region
(D) The meandering index of tracked macrophages in PBS injected tail fin region.
(E) The meandering index of tracked neutrophils in PBS injected tail fin region.
Data (mean ± SD) were combined from three independent experiments with 5 or 6 fish in *tlr2^+/+^* or *tlr2^-/-^* group. An unpaired, two-tailed t-test was used to assess significance. ns, non-significant. Scale bar: 100 µm; Sample size (n): 347, 273; 170, 134 (B, D, and C, E).
